# Supplementary material for: Crocodilepox Virus Evolutionary Genomics Supports Observed Poxvirus Infection Dynamics on Saltwater Crocodile (Crocodylus porosus)
Source: Viruses. 2019 Dec 2;11(12):1116. doi: 10.3390/v11121116 (PMC6950651; doi:10.3390/v11121116)
Supplement: Supplementary file 1 [file viruses-11-01116-s001.pdf]

Crocodilepox virus evolutionary genomics supports observed poxvirus infection dynamics on saltwater crocodile (*Crocodylus porosus*)

Subir Sarker,[a,*] Sally R Isberg,[b,c] Jasmin L Moran,[b] Rachel De Araujo,[d] Nikki Elliott,[d] Lorna Melville,[d] Travis Beddoe,[e] Karla J Helbig,[a,*]

[a]Department of Physiology, Anatomy and Microbiology, School of Life Sciences, La Trobe University, Bundoora, VIC 3086, Australia
[b]Centre for Crocodile Research, Noonamah, NT, Australia
[c]School of Psychological and Clinical Sciences, Charles Darwin University, Darwin, NT, Australia
[d]Berrimah Veterinary Laboratory, Northern Territory Government, Darwin, Northern Territory, Australia
[e]Department of Agriculture Sciences, School of Life Sciences, La Trobe University, Bundoora, VIC 3086, Australia

[*]**Authors for correspondence:**

Dr Subir Sarker, Department of Physiology, Anatomy and Microbiology, School of Life Sciences, La Trobe University, Bundoora, VIC 3086, Australia, email: s.sarker@latrobe.edu.au; phone: +61 3 9479 2317; fax: +61 3 9479 1222.

Associate Professor Karla Helbig, Department of Physiology, Anatomy and Microbiology, School of Life Sciences, La Trobe University, Bundoora, VIC 3086, Australia, email: k.helbig@latrobe.edu.au; phone: +61 3 9479 6650; fax: +61 3 9479 1222.

		1	2	3	4	5	6	7	8	9	10	11	12	13	14	15	16
F3a [MK903858]	1		0.00	0.00	0.00	0.00	0.00	0.00	0.00	0.01	0.01	0.01	0.01	0.01	0.01	0.01	0.01
F3b [MK903859]	2	99.89		0.00	0.00	0.00	0.00	0.00	0.00	0.01	0.01	0.01	0.01	0.01	0.01	0.01	0.01
F3c [MK903860]	3	99.66	99.71		0.00	0.00	0.00	0.00	0.00	0.01	0.01	0.01	0.01	0.01	0.01	0.01	0.01
F2d [MK903856]	4	99.72	99.75	99.57		0.00	0.00	0.00	0.00	0.01	0.01	0.01	0.01	0.01	0.01	0.00	0.01
F2a [MK903853]	5	99.59	99.61	99.47	99.80		0.00	0.00	0.00	0.01	0.01	0.01	0.01	0.01	0.00	0.00	0.01
F2e [MK903857]	6	98.85	98.87	98.75	99.06	99.24		0.00	0.00	0.00	0.01	0.00	0.00	0.01	0.00	0.00	0.01
F5a [MK903861]	7	98.92	98.95	99.20	98.89	99.04	98.43		0.00	0.01	0.01	0.01	0.01	0.01	0.01	0.01	0.01
F5b [MK903862]	8	99.08	99.11	99.30	99.06	99.07	98.40	99.72		0.01	0.01	0.01	0.01	0.01	0.01	0.01	0.01
F1a [MK903850]	9	98.97	98.97	98.79	99.03	98.98	98.54	98.41	98.48		0.00	0.00	0.00	0.00	0.01	0.01	0.01
F1b [MK903851]	10	99.10	99.14	98.96	99.16	99.18	98.48	98.74	98.80	99.38		0.00	0.00	0.00	0.00	0.00	0.01
F1c [MK903852]	11	98.51	98.51	98.45	98.54	98.52	98.46	98.09	98.14	99.36	98.93		0.00	0.00	0.00	0.01	0.01
SwCRV-1 F1d [MG450915]	12	99.18	99.11	98.93	99.18	99.06	98.34	98.44	98.65	99.62	99.45	99.05		0.00	0.01	0.01	0.01
F2b [MK903854]	13	99.05	99.10	98.95	99.12	99.10	98.43	98.71	98.81	99.17	99.65	98.71	99.25		0.01	0.01	0.01
F4a [MK903863]	14	97.98	97.90	97.71	98.00	97.89	97.22	97.27	97.47	97.81	97.88	97.36	97.96	97.78		0.00	0.00
SwCRV-2 F1e [MG450916]	15	97.86	97.88	97.68	97.96	97.88	97.23	97.26	97.40	97.77	97.84	97.35	97.79	97.73	99.73		0.00
F2c [MK903855]	16	97.64	97.66	97.50	97.68	97.78	97.18	97.35	97.37	97.49	97.81	97.03	97.45	97.76	99.21	99.22	

min max

Figure S1: Pairwise comparison on 16 genome sequences of SwCRV, including 14 SwCRV genomes isolated in this study. Upper diagonal indicates the distance between two sequences, and the lower diagonal shows percentage identity between two sequences.

Table S1. Predicted recombination events among SwCRV genomes sequenced in this study

	**Breakpoint positions**														
	**In alignment**		**In recombinant sequence(s)**					**Detection methods**							
**Events**	**Begin**		**Begin**	**End**	**Recombinant Sequence(s)**	**Minor Parental Sequence(s)**	**Major Parental Sequence(s)**	**RDP**	**GENECONV**	**Bootscan**	**Maxchi**	**Chimaera**	**SiSscan**	**3Seq**
RE1	28110	51457	27930	51257	F2b, F1a-F1c, F1d	F3a-F3c	F4a, F1e	4.62E-106	3.46E-164	7.82E-74	7.60E-48	9.34E-48	6.11E-34	6.22E-14
RE2	6956	25638	6700	25316	F3a-F3b, ^F3c, F2a, F2d, F2e	F2c	F5a-F5b	1.14E-121	7.04E-123	1.54E-34	1.15E-37	6.38E-39	5.64E-22	6.22E-14
RE3	168605	170188	168001	169104	F5a, ^F5b	Unknown	F3c	1.63E-77	5.61E-74	1.52E-77	2.10E-22	1.84E-22	3.68E-22	1.24E-13
RE4	25639*	39486	24905*	38711	F5a, ^F5b	Unknown	F2a, F2d, F2e	8.96E-57	1.12E-62	5.96E-63	1.93E-27	1.29E-27	-	1.24E-13
RE5	24733	25629*	24521	25343*	F3a-F3c, ^F2c, F2d	Unknown	F1a-F1c, F1d-2, F4a	8.41E-62	6.26E-55	3.71E-65	1.95E-25	4.29E-25	-	9.17E-52
RE6	168516	170178	165643	167053	F1e, F4a	F3c, F2a, F2d, F2e	F1a, F1d	1.17E-51	7.29E-57	1.31E-58	3.36E-23	6.07E-16	9.78E-33	6.22E-14
RE7	179026	6955*	178565	6833*	F2a, ^F2d, F2e	F1d	F3a-F3c, F5a-2	7.80E-57	1.89E-29	4.33E-22	8.35E-26	6.12E-27	8.58E-19	1.31E-03
RE8	166912	173988	166684	173168	^F1b	F2b	F1a, F1c	1.81E-49	1.68E-47	1.80E-49	3.06E-24	5.67E-24	1.18E-20	6.22E-14
RE9	178352	184700	178215	184552	F1d, F2a, F2d, F4a	Unknown	F1a-F1c	2.52E-37	6.18E-46	2.06E-44	1.42E-15	1.34E-15	5.27E-14	6.22E-14
RE10	39487*	105464	39148*	105080	F5a, ^F3c	F3a-F3b	F5b	1.13E-37	4.09E-37	1.14E-24	1.04E-20	7.19E-21	4.06E-30	6.22E-14
RE11	174248	178988*	172273	176999*	F2a, F2d, ^F2e	F1e, F4a	F5b	7.71E-34	5.53E-36	1.40E-24	8.06E-15	5.17E-15	2.53E-02	6.22E-14
RE12	7918	21690	7794	21519	^F2b	Unknown	F1a-F1c, F1d-2, F4a	4.31E-26	2.96E-34	3.47E-33	2.26E-07	5.23E-18	8.23E-25	8.39E-17
RE13	43728	51457*	43552	51279*	^F3a, F3b-F3c, F2b	Unknown	F2a, F2d, F2e, F5b	1.14E-32	1.48E-34	1.13E-32	2.55E-11	7.30E-11	8.76E-07	6.22E-14
RE14	171517*	176653	171017*	176137	F3a, ^F3b	F1e, F4a	F3c, F5a-2	1.32E-28	3.69E-29	1.33E-28	1.77E-12	4.09E-12	-	6.22E-14
RE15	173989*	178328	173244*	177566	F2b	F3a-F3c, F5a-2	F1a-3	1.40E-28	-	1.80E-03	7.73E-09	2.56E-10	2.36E-08	1.24E-13
RE16	63497	71860	63284	71647	F1a-3, F1d	F3a-2, F5a-2, F2a, F2d, F2e	F2b	5.17E-21	1.87E-17	1.72E-07	1.02E-06	1.00E-06	-	6.22E-14
RE17	151594	169174	151372	168939	^F2b	Unknown	F1a, F1c, F1d	2.91E-20	6.10E-05	-	5.99E-03	1.42E-02	-	6.22E-14
RE18	184701*	1658	181555*	1547	^F1e	Unknown	F4a	2.89E-19	7.47E-12	1.58E-18	3.23E-09	2.92E-09	1.45E-05	6.22E-14
RE19	36812	40388	34150	37726	^F4a	F2b	Unknown	-	6.33E-16	-	1.81E-05	1.77E-05	9.85E-29	2.05E-10

RE20	49569	50176	49060	49667	^F1a, F1c	F2a, F2d, F2e, F1e	F1b	-	1.56E-13	-	2.94E-03	2.94E-03	-	3.22E-07
RE21	173382	184700*	170257	181554*	F4a, ^F1e	Unknown	F1a-3	2.42E-13	1.58E-13	7.53E-12	1.25E-12	5.59E-04	9.04E-08	1.87E-13
RE22	131012	142784	130796	142559	^F1b, F2b	F2c	F1a, F1c, F1d	2.84E-09	4.42E-10	2.88E-09	1.25E-05	2.00E-04	6.08E-19	3.96E-08
RE23	36812*	38627	33975*	35790	^F1e, F2c	F2b	Unknown	5.13E-03	1.09E-07	-	1.60E-02	-	1.40E-29	2.63E-04
RE24	174281*	175339	173751*	174795	^F1a, F1b-3, F1d, F2c	F1e	F3c, F5a-2	1.26E-06	1.93E-08	-	3.98E-08	2.12E-03	8.40E-10	1.37E-07

* = The actual breakpoint position is undetermined (it was most likely overprinted by a subsequent recombination event).
^ = The recombinant sequence may have been misidentified (one of the identified parents might be the recombinant)
- = No significant P-value was recorded for this recombination event using this method.

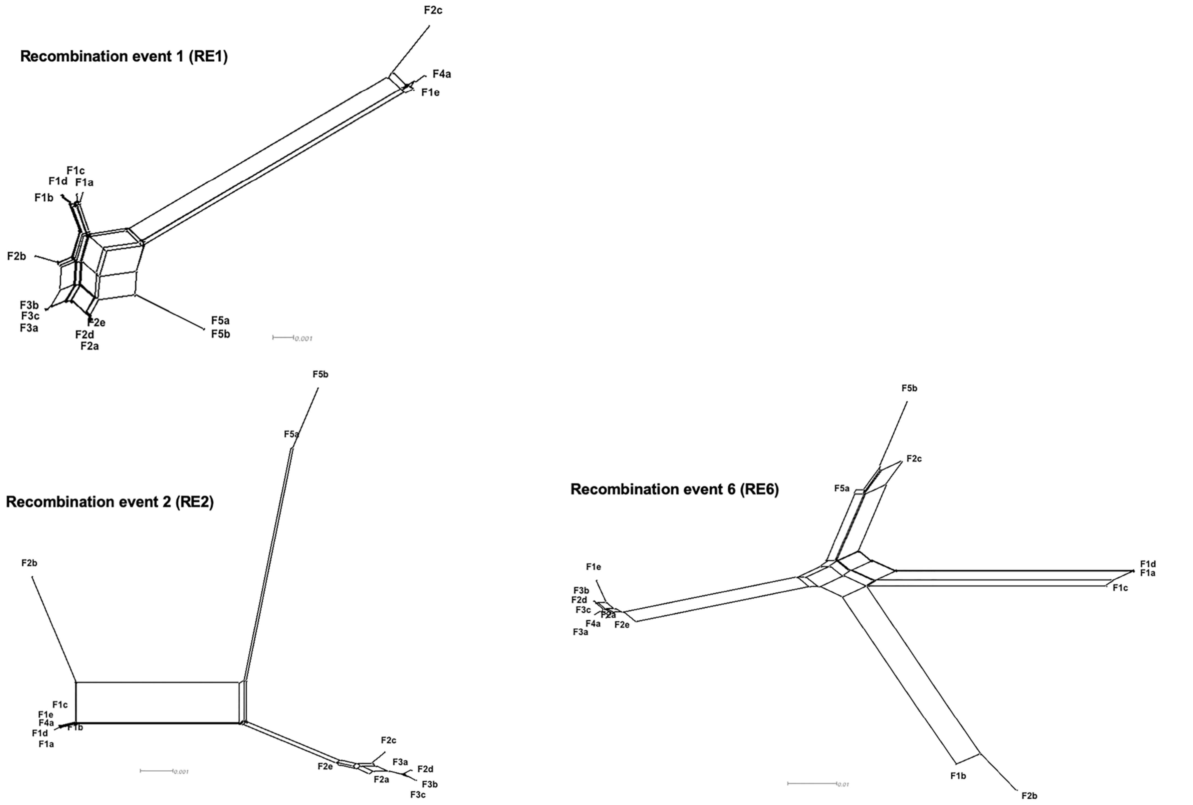

Figure S2: NeighborNet trees were generated using selected recombination segments (recombination events RE1, -RE2 and -RE6).

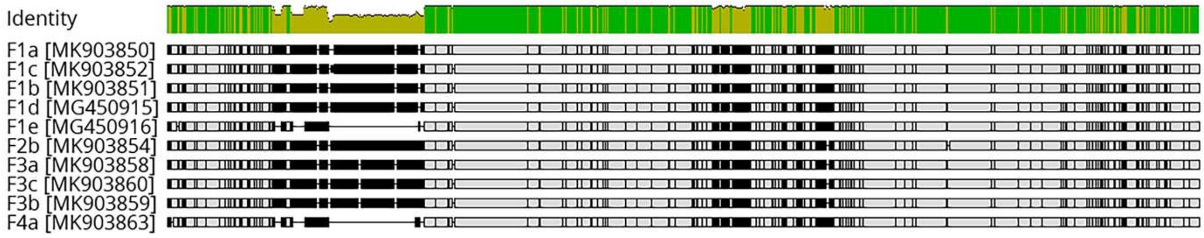

Figure S3: Consensus identity graph representing the mean pairwise identity over all pairs in the column (RE1 segment); where, colour green: 100% identity, greeny-brown: at least 30% and under 100% identity and red: below 30% identity. Vertical black lines highlighting the disagreement of individual SwCRV sequence in comparison to consensus.

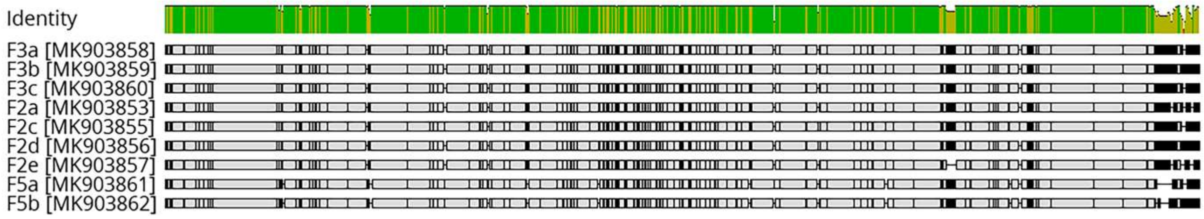

Figure S4: Consensus identity graph representing the mean pairwise identity over all pairs in the column (RE2 segment); where, colour green: 100% identity, greeny-brown: at least 30% and under 100% identity and red: below 30% identity. Vertical black lines highlighting the disagreement of individual SwCRV sequence in comparison to consensus.

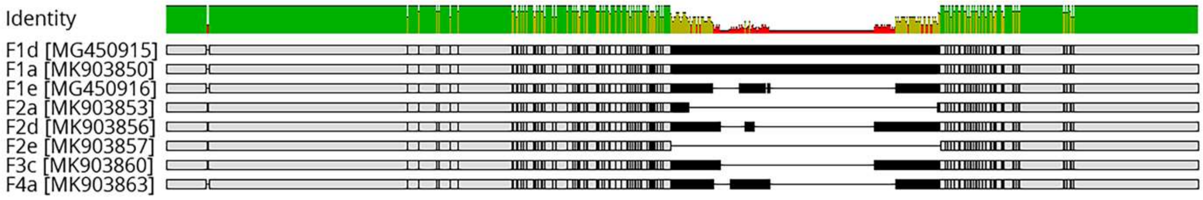

Figure S5: Consensus identity graph representing the mean pairwise identity over all pairs in the column (RE6 segment); where, colour green: 100% identity, greeny-brown: at least 30% and under 100% identity and red: below 30% identity. Vertical black lines highlighting the disagreement of individual SwCRV sequence in comparison to consensus.

		1	2	3	4	5	6	7	8	9	10
F1a [MK903850]	1		0.00	0.00	0.00	0.02	0.01	0.01	0.01	0.01	0.02
F1c [MK903852]	2	99.81		0.00	0.00	0.02	0.01	0.01	0.01	0.01	0.02
F1b [MK903851]	3	99.52	99.59		0.00	0.02	0.00	0.01	0.01	0.01	0.02
F1d [MG450915]	4	99.52	99.60	99.99		0.02	0.00	0.01	0.01	0.01	0.02
F1e [MG450916]	5	87.37	87.40	87.06	87.07		0.02	0.02	0.02	0.02	0.00
F2b [MK903854]	6	99.08	99.16	99.46	99.46	86.90		0.00	0.00	0.00	0.02
F3a [MK903858]	7	98.98	99.01	99.32	99.32	86.87	99.60		0.00	0.00	0.02
F3c [MK903860]	8	98.98	99.01	99.32	99.32	86.87	99.60	100.00		0.00	0.02
F3b [MK903859]	9	98.98	99.01	99.32	99.32	86.87	99.60	100.00	100.00		0.02
F4a [MK903863]	10	87.56	87.59	87.27	87.28	99.55	87.10	87.04	87.04	87.04	

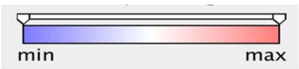

Figure S6: Pairwise comparison on RE1 segment among the SwCRV sequences involved. Upper comparison gradient indicated the distance between two sequences, and lower comparison gradient indicated percentage identity between two sequences.

		1	2	3	4	5	6	7	8	9
F3a [MK903858]	1		0.00	0.00	0.00	0.00	0.00	0.00	0.01	0.02
F3b [MK903859]	2	99.82		0.00	0.00	0.00	0.00	0.00	0.01	0.02
F3c [MK903860]	3	99.99	99.82		0.00	0.00	0.00	0.00	0.01	0.02
F2a [MK903853]	4	99.66	99.48	99.65		0.00	0.00	0.00	0.01	0.01
F2c [MK903855]	5	99.54	99.36	99.53	99.27		0.00	0.00	0.01	0.02
F2d [MK903856]	6	99.73	99.89	99.72	99.49	99.44		0.00	0.01	0.02
F2e [MK903857]	7	98.25	98.08	98.25	98.59	98.30	98.08		0.01	0.01
F5a [MK903861]	8	96.42	96.26	96.42	96.48	96.21	96.22	95.30		0.00
F5b [MK903862]	9	96.88	97.02	96.88	96.70	96.66	96.99	95.42	98.96	

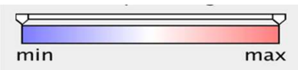

Figure S7: Pairwise comparison on RE2 segment among the SwCRV sequences involved. Upper comparison gradient indicated the distance between two sequences, and lower comparison gradient indicated percentage identity between two sequences.

		1	2	3	4	5	6	7	8
F1d [MG450915]	1		0.00	0.14	0.10	0.13	0.09	0.13	0.12
F1a [MK903850]	2	100.00		0.14	0.10	0.13	0.09	0.13	0.12
F1e [MG450916]	3	74.40	74.40		0.00	0.01	0.00	0.00	0.02
F2a [MK903853]	4	68.49	68.49	88.83		0.00	0.00	0.00	0.00
F2d [MK903856]	5	75.29	75.29	93.90	88.24		0.00	0.00	0.01
F2e [MK903857]	6	67.41	67.41	86.70	97.62	86.13		0.00	0.00
F3c [MK903860]	7	74.92	74.92	93.42	89.11	99.02	86.99		0.00
F4a [MK903863]	8	76.69	76.69	96.57	87.51	92.69	85.42	92.15	

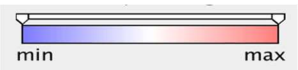

Figure S8: Pairwise comparison on RE6 segment among the SwCRV sequences involved. Upper comparison gradient indicated the distance between two sequences, and lower comparison gradient indicated percentage identity between two sequences.
